# Syphilis in Maria Salviati (1499–1543), Wife of Giovanni de’ Medici of the Black Bands

**DOI:** 10.3201/eid2606.180786

**Published:** 2020-06

**Authors:** Antonio Fornaciari, Raffaele Gaeta, Simona Minozzi, Valentina Giuffra

**Affiliations:** University of Pisa, Pisa, Italy

**Keywords:** syphilis, renaissance, Florence, Italy, Medici, sexuality, bacteria, treponematosis, sexually transmitted infections, Maria Salviati

## Abstract

Researchers from the Division of Paleopathology of Pisa University (Pisa, Italy) exhumed the well-preserved skeleton of Maria Salviati (1499–1543), wife of Giovanni de’ Medici, named “Giovanni of the Black Bands,” in Florence in 2012. Many lytic lesions had affected the skull of Maria on the frontal bone and on the parietal bones. These lesions are pathognomonic for syphilis. An ancient diagnosis of syphilis for Maria Salviati does not emerge from the historical sources, although the symptoms manifested in her last years of life are compatible with a colorectal localization, including severe hemorrhages, caused by syphilitic infection. The case of Maria Salviati can be compared with those of other famous Italian noblewomen of the Renaissance, such as Isabella of Aragon (1470–1524) and Maria of Aragon (1503–1568). Paleopathology made it possible to directly observe a “secret illness” to which noblewomen were susceptible as a result of the sexual conduct of their husbands.

Syphilis today is a reemerging infectious disease that affects not only the developing countries but also the Western world. In recent years, a new increase has occurred in the incidence of sexually transmitted diseases, among which syphilis is one of the most common (*1*). Especially in the United States, the rates of primary and secondary syphilis have increased since 2000–2001. A total of 27,814 syphilis cases were reported in 2016 (*2*). During 2015–2016, the US syphilis rate increased by 17.6%, reaching 8.7 cases/100,000 population, the highest rate reported since 1993 (*2*). Europe experienced a similar trend; in 2016, a total of 29,365 confirmed syphilis cases were reported in 28 countries, a rate of 6.1 cases/100,000 population. The highest rates (cases/100,000 population) in Europe were observed in the United Kingdom (9.9), Malta (9.2), Iceland (9.0), and Germany (8.7) (*3*).

Venereal syphilis is a treponematosis caused by the bacterium *Treponema pallidum* subsp. *pallidum*, the most widespread disease among the 4 treponematoses. Pinta (*T. carateum* infection) is spread only in tropical areas of the Americas, yaws (*T. pallidum* subsp. *pertenue* infection) in humid tropical and subtropical regions, and bejel (*T. pallidum* subsp. *endemicum* infection) in arid-temperate and subtropical rural areas. All these diseases involve the human bone, with the exception of pinta. Three clinical stages are typical of venereal syphilis: the primary stage is a painless lesion (chancre) on the genitals, which heals in 2–6 weeks; some months later, the secondary stage is characterized by a widespread skin rash; and several years later, the tertiary stage involves different organs, including the skeleton (*4*).

Venereal syphilis first emerged in Europe at the end of the 15th century, as a result of the sexual and social behavior of the time (*5*). Soon after the disease’s arrival, its sexually transmitted nature was recognized, becoming a mark of immoral behavior (*6*); however, the social implication of syphilis was not the same for men and women, especially in the aristocracy (*7*). In fact, the sexual conduct of the noblemen and the possible infectious diseases that followed were not subject to moral censorship; instead, there was severe moral judgement for venereal diseases in noblewomen (*7*–*9*). Some paleopathological cases have indicated the impact of syphilis on the aristocratic classes of the Renaissance (*10*–*12*). Paleopathology offers a source for increasing the diagnosis of infection in the past and for understanding the social and cultural impact of infectious diseases in previous populations. The models obtained can be compared with what happens today with emerging and reemerging diseases and can serve to refine the systems of prevention and fight against future infection outbreaks (*13*). The study of the skeletal remains of Maria Salviati (1499–1543), wife of Giovanni de’ Medici, which revealed lesions typical of third-stage syphilis, has enabled us to understand the dynamics of the infection in one of the most famous families of the Renaissance and to examine the perception of the illness in 16th century Europe.

## Syphilis in the 16th Century

After the 1494 invasion of Italy by the troops of Charles VIII, King of France (1470–1498), venereal syphilis had a pandemic spread in Italy and in Europe (*9*). The origin of the disease remains one of the greatest issues in the history of medicine and is still discussed by scholars. One theory suggests the disease originated in the Americas and was introduced by Columbus’ crew returning to Europe from the New World in 1493. According to a second theory, syphilis previously existed in the Old World but went unrecognized until the late 15th century, when there was increased prevalence and virulence of the disease (*14*). In the past few years, further paleopathologic evidence has indicated the presence of the disease in Europe before 1492 (*15*,*16*). However, the transmission at the end of the 15th century is undeniable and can be explained in the light of the wider sociocultural context of the period (*17*,*18*). 

From the late 15th to mid-16th centuries, Italy was a great field of war and an ideal social environment for the spread of the disease. In the Renaissance, before the advent of the Catholic Counter-Reformation, Italy experienced an increase in the volume of trade exchanges, new contacts between populations, migration from other countries, and, above all, a time of greater sexual liberty (*5*,*19*). The activity of prostitution among the troops and the civilians in the towns, as well as the opportunities for extramarital sex created by the permanence of armies, generated a perfect basis for the spread of syphilis (*7*,*9*). Because of its sexual connotation, the disease embodied the concept of divine ill-punishment, which, as an archetype, pervaded the popular religious sensibility of European populations of the early modern age (*20*). The early pandemic and violent phase of syphilis, before the classical chronicization to three stages, had an impressive impact on European society (*5*,*21*). 

The first physician observing syphilis was Alessandro Benedetti, field doctor of the Italian confederate army fighting against the French during the battle of Fornovo in 1495 (*22*). Girolamo Fracastoro (1548) successfully coined the name of the disease in his famous poem “Syphilis sive morbus gallicus [Syphilis or the French disease]” (*23*). Contemporary physicians described the manifestation of the disease, consisting in the appearance of an ulceration on the penis, followed by pustules and sores all over the face and body with joint pain and pruritus. The doctors quickly acknowledged that the infection had been transmitted through sexual intercourse (*17*). After the most aggressive first phase of the “new” disease, syphilis quickly changed from an acute and debilitating disease into a less severe chronic infection, probably because the selection of the less virulent strains represented an evolutionary advantage for the pathogen (*21*). 

Syphilis was rife in all social classes and affected many members of the aristocracy. Many noblemen undertook military careers as captains of mercenary troops, which typically involved extramarital affairs, not only with regular lovers but also, and frequently, with prostitutes (*24*). Famous are the cases of Cesare Borgia (1476–1507), son of Pope Alexander VI, who had to wear a leather mask covering half of his face, which had been disfigured by syphilis in his later years (*25*), and of Francesco II Gonzaga (1466–1519), Marquis of Mantua, who had a form of tertiary syphilis (*23*). Evidence that the disease was widespread in the 16th century aristocratic classes is also demonstrated by paleopathology. The cases of Maria of Aragon, Marquise of Vasto (1503–1568) (*10*), Vespasiano Gonzaga, Duke of Sabbioneta (1531–1591) (*11*), and Cardinal Giulio Della Rovere (1533–1578) (*12*) are some of the most famous Renaissance figures for whom syphilis was diagnosed.

## Brief Biography and Nosography of Maria Salviati

Maria Salviati, daughter of Lucrezia de’ Medici and Jacopo Salviati and granddaughter of Lorenzo the Magnificent, was born in Florence in 1499. Her marriage to Giovanni de’ Medici (1498–1526) took place in 1516. Giovanni died of gangrene and septicemia (*26*) on November 30, 1526, complications resulting from an injury and amputation of his right leg after the Battle of Governolo, near Mantua, leaving his wife a widow at the age of 27. Maria never remarried. Cosimo, the son of Maria and Giovanni, was called to govern Florence after the death of Duke Alessandro de’ Medici (1537), giving rise to the Grand Ducal Medici branch, which ruled Tuscany until 1737. 

Archival research by Gaetano Pieraccini was able to reveal important information about Maria Salviati, with particular regard to the last years of her life (*24*). Until 1540, she enjoyed good health, except for a brief episode of fever of unknown origin recorded in 1517. In the last 3 years of her life, from May 1541 to her death on December 29, 1543, many symptoms of severe illness were described in letters sent by Andrea Pasquali, the court physician, to Duke Cosimo, including abundant recurring proctorrhagias (bleeding from the rectum from ½ to 3 libras of blood [i.e., 180 g to ≈1 L]), rectal and perianal ulcers, headaches, and abdominal colic. The letters also report “chronic weakness…, shortness of breath, frequent lipothymic episodes, severe syncopes, cold extremities, vomit and agitation…” and note “the pulse was deeply reduced, the frequency increased” (*24*). The symptoms were certainly the expression of a severe anemia caused by chronic leakages of blood. 

**Figure 1:**
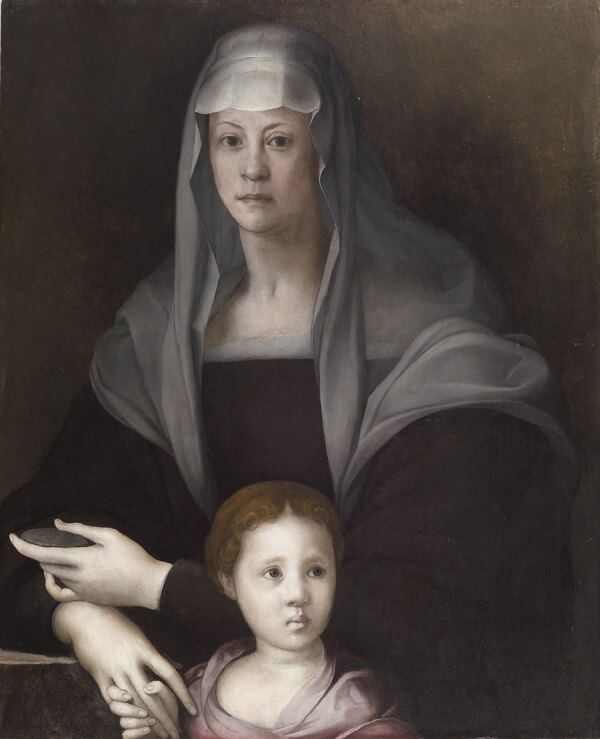
Portrait of Maria Salviati and Giulia de’ Medici depicted by Pontormo (Jacopo Carucci) in 1537 c. Oil on panel. 34.65 × 28.07 in. (88 × 71.3 × 1 cm). (The Walters Art Museum, Baltimore.)

**Figure 2:**
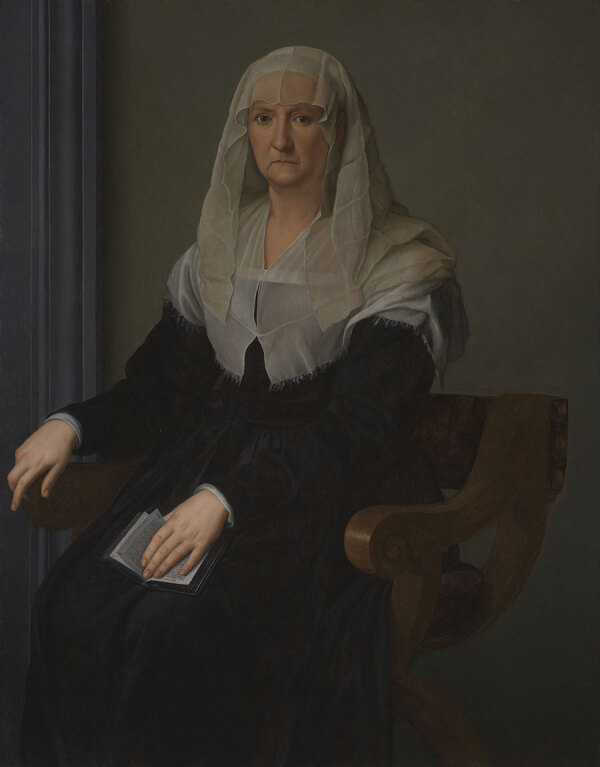
Portrait of an Elderly Lady (Maria Salviati) depicted by Agnolo Bronzino in 1542–1543 c. Oil on panel. 50 × 39.4 in. (127 × 100 cm). (San Francisco, The Fine Arts Museum of San Francisco, Gift of Mr. Samuel H. Kress, 53670. Image courtesy the Fine Arts Museums of San Francisco).

The portraits of Maria Salviati clearly show the significant changes that occurred in her aspect, marking the progress in terms both of age and of illness. A portrait by Jacopo Pontormo (Figure 1), painted in 1537 and preserved at the Uffizi Museum, shows Maria as a beautiful lady, still young; but only 6 years later, in a portrait by Bronzino (Figure 2), she appears as a very old woman. Rather than to the artistic choices of the painter, this transformation seems to be strongly related to the accelerating physical decay of Maria, which was probably connected to the illness in the last years of her life.

## Historical, Archeological, and Taphonomic Background

**Figure 3:**
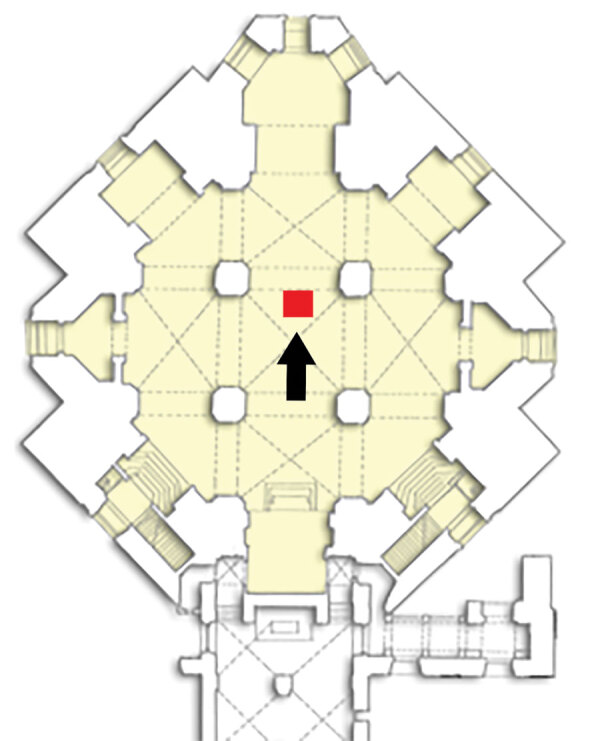
Plan of the crypt of the Medici Chapel with the position of the tomb of Maria Salviati. (Archive of the Division of Paleopathology. University of Pisa.)

Almost all the bodies of the Medici, and also that of Maria Salviati, were embalmed before burial (*24*), but it was not until the mid-19th century that they were given a definitive grave location. Until the 19th century, the bodies of the Medici of the Grand Duchy dynasty were preserved in wooden coffins inside the 2 sacristies of the Basilica of San Lorenzo in Florence. During 1857–58, the Gran Duke of Tuscany Leopold II of Lorrain arranged the bodies of the Medici in the crypt of the Medici Chapel to give a proper and dignified burial place to the founders of the Grand Duchy of Tuscany (*27*). The body of Maria Salviati, identified in 1857 thanks to the presence of a copper epigraph on the coffin (*28*), was deposed, together with that of her husband Giovanni “of the Black Bands,” in a tomb at the center of the crypt floor (Figure 3). We have a description of this event given by Luigi Passerini-Rilli, director of the State Archive of Florence and responsible for the recognition of the bodies of the Grand Dukes (*28*): “The body, although reduced to almost a skeleton in the face, was however very well preserved in the other parts.... The head lay on two bricks.... The clothing that covered it resembled that of a nun, i.e. a black cloth, but eaten by the moths: the leftovers of the wimple were still discernible, though the veil that at first covered the head, was worn....” 

**Figure 4:**
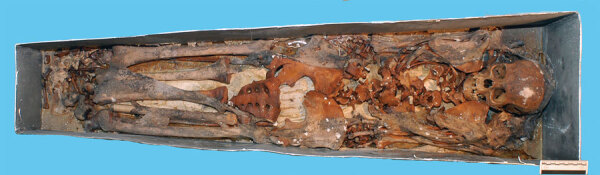
The skeletal remains of Maria Salviati at exhumation in 2012. (Archive of the Division of Paleopathology. University of Pisa.)

In 1946, Gaetano Pieraccini and the anthropologist Giuseppe Genna conducted an exhumation (*29*); they heavily manipulated the skeletal remains of Maria with the removal of residual soft tissues before reburial in a small zinc coffin (*27*). On this occasion, they made a plaster cast of the skull, which is now in the Museum of Anthropology of Florence. In November 2012 the last exhumation took place, under the management of the Division of Paleopathology of the University of Pisa, during some architectural checks of the stability of the floor of the Chapel. The paleopathologists found that the remains of Maria were in excellent state of preservation, unconnected in a small zinc coffin bearing an epigraph with her name (Figure 4).

## Anthropologic and Paleopathologic Study

**Figure 5:**
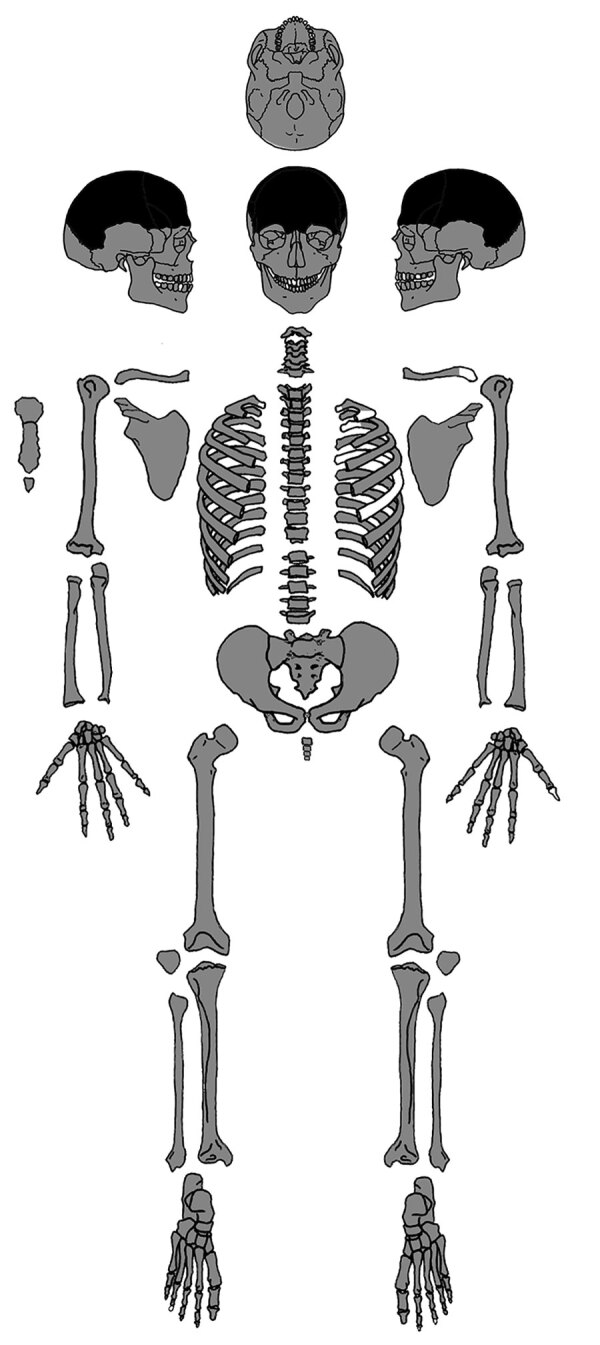
Present-day bones of the skeleton of Maria Salviati (gray). Distribution of lesions (black). (Archive of the Division of Paleopathology. University of Pisa.)

**Figure 6:**
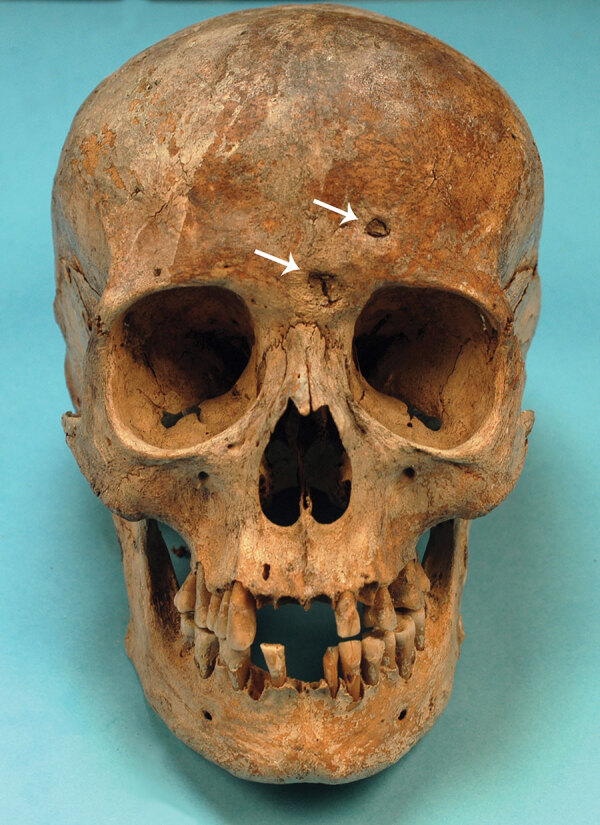
The skull of Maria Salviati in frontal view. Cavitations on the frontal bone are apparent. (Archive of the Division of Paleopathology. University of Pisa.)

**Figure 7:**
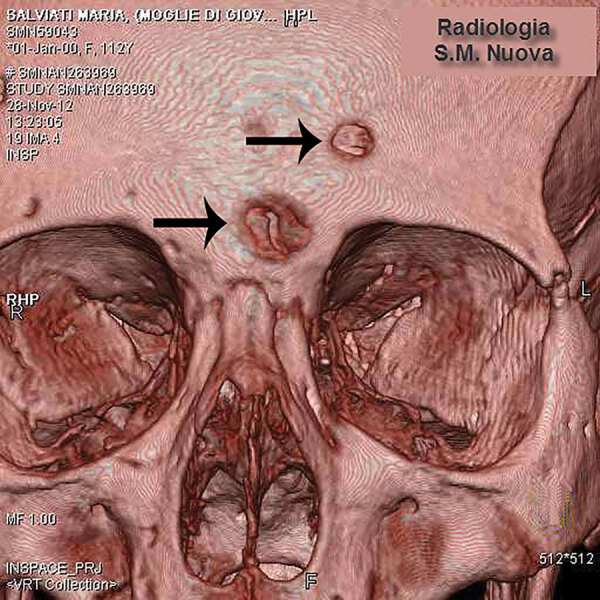
Volume rendering of the skull of Maria Salviati. Two destructive osteolytic inflammatory processes, in advanced reparative phase (circumvallate cavitations), are apparent. (Archive of the Division of Paleopathology. University of Pisa.)

**Figure 8:**
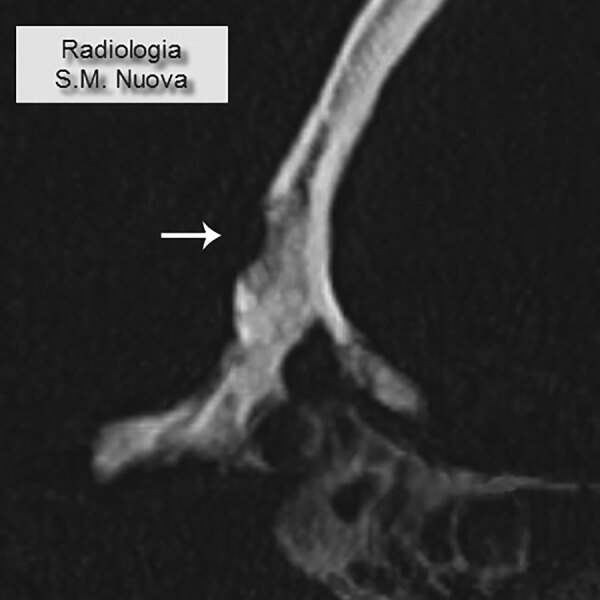
Computed tomography scan of frontal bone with an osteolytic lesion with sclerotic walls (circumvallate cavitations). (Archive of the Division of Paleopathology. University of Pisa.)

**Figure 9:**
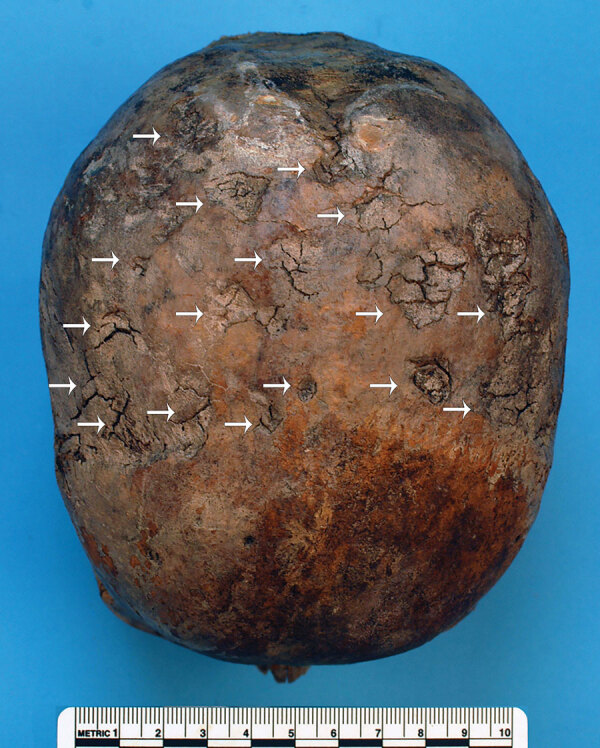
Parietal bones of Maria Salviati, showing several radial scars typical of tertiary syphilis. (Archive of the Division of Paleopathology. University of Pisa.)

**Figure 10:**
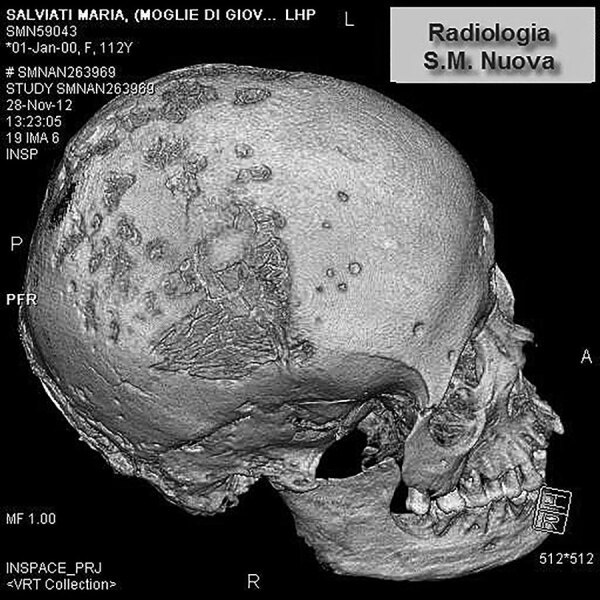
Volume rendering of the skull of Maria Salviati, showing contemporary presence of lytic (superficial cavitations) and reparative lesions (radial scars).(Archive of the Division of Paleopathology. University of Pisa.)

We examined the nearly complete skeleton of Maria Salviati macroscopically (Figure 5) and performed radiographic and computed tomography (CT) scans. We compared the skull bone recovered during exhumation with the skull cast, and they showed the same lesions. The anthropologic study of the skeleton revealed a female individual (*30*), 40–45 years of age (*31*–*34*), with a stature of 1.56 m (*34*). Maria had severe periodontal disease, as evidenced by the resorption of alveolar edges and an abscess at the buccal portion of the third right maxillary molar. Nonpenetrating caries affected 10 teeth, of which 4 were mandibular and 6 maxillary. The poor dental health of Maria is consistent with that of the other members of the Medici family (*35*). We detected many lytic lesions on the skull. Two circular ectocranial depressions are visible on the frontal bone, on the glabella and above the left supraorbital ridge. They are irregularly elliptical in shape, measuring ≈1 × 0.7 cm and 0.5 × 0.4 cm, respectively, with a central destructive focus and a reactive compact bone formation on the margins (Figure 6). These frontal bone lesions are consistent with 2 destructive osteolytic inflammatory processes, in advanced reparative phase, as clearly revealed by CT examination (Figures 7, 8). Furthermore, the cranial vault on the parietal bones shows several osteolytic lesions in the form of circumvallate depressed areas with fine scar lines radiating inside the shallow depressions (Figures 9, 10). CT examination confirms the lytic and reparative nature of the lesions, which are morphologically similar to internodular stellate depressions. We observed no other lesions in the postcranial bones macroscopically, by radiograph, or by CT scan. The presence of strong bone reaction excludes metastatic osteolytic carcinoma, multiple myeloma, tuberculosis, and fungal bone infections (*36*,*37*). The presence of superficial circumvallated cavitations with radial scars is pathognomonic of cranial syphilis (caries sicca) (*38*).

## Discussion

The combination of crater-like lesions, such as circumvallate cavitations on the frontal bone (phase 4 in the Caries Sicca sequence depicted in Hackett [*38*]), and circumvallate cavitations with radial scars on the parietal bones (phase 5 in the Caries Sicca sequence [*38*]) is pathognomonic for tertiary syphilis. Pathography attests that Maria had abundant recurring proctorrhagias. This symptom strongly suggests tertiary syphilis with cranial and possible colorectal localization. Apart from proctorrhagias, the other symptoms, such as frequent fever and headache, abdominal colic, rectal and perianal ulcers, reported by the written sources are very compatible with tertiary syphilis (*4*,*39*,*40*) but were not attributed to syphilis by the physicians of that time nor by the nosographic scholars in recent times (*24*). Nevertheless, rectal syphilis is a rare disease that usually shows proctitis with perianal ulcers but lacks pathognomonic clinical symptoms, making diagnosis difficult. In clinical medicine, anal syphilis could be easily misdiagnosed as cancer or advanced stage hemorrhoids, upholding the reputation of syphilis as the “great mimicker” (*41*).

One hypothesis is that the contemporary physicians might have correctly diagnosed and recognized Maria Salviati’s disease but concealed the sexual component of the infection. Another hypothesis is that Maria Salviati, who never allowed the physicians to inspect her genitals (*24*), might have hidden the symptoms of her disease out of modesty. A further explanation, based on political reasons, is that the mother of Duke Cosimo I could not appear to be affected by venereal syphilis, to avoid corrupting the image of the Medici family that Cosimo was laboriously trying to promote among the royal rank. The fact that in her last years of life Maria was always portrayed with a veil might indicate her intention to hide the luetic skin lesions.

Syphilis is likely to have been more widespread among the noblewomen of the Renaissance than is attested by the written sources. Paleopathology has in some cases revealed some hidden illnesses of the Italian noblewomen, as in the case of Isabella of Aragon, Duchess of Milan (1470–1524), and Maria of Aragon (1503–1568), Marquise of Vasto and wife of the governor of Milan Alfonso of Avalos (1502–1546). Isabella was probably affected by syphilis, despite the absence of bone lesions on her skeletal remains. The syphilitic infection was diagnosed indirectly, on the basis of the paleopathologic analyses performed on her teeth (*42*). On the buccal surfaces of the teeth, Isabella showed a strong abrasion caused by pumice powder and toothpicks of cuttlebone that she used to remove the blackish patina produced by her mercurial therapy. In fact, energy-dispersive spectroscopic analysis of the dark material detected a massive presence of mercury, largely employed in the treatment of syphilis since the early 16th century in the form of salves, fumigations, and ointments (*43*,*44*). The care given to Isabella of Aragon clearly demonstrates her willingness to erase the evident traces of chronic mercury intoxication caused by the antisyphilitic therapy. However, no references to syphilis are reported in the written documents about the life of Isabella. In the artificial mummy of Maria of Aragon, the histologic, immunohistochemical, and ultrastructural study of a cutaneous ulcer of the left arm led to the direct identification of *Treponema pallidum* and the diagnosis of tertiary venereal syphilis (*10*). The biographic sources report that Maria of Aragon periodically spent time at the Agnano Baths, near Naples (*45*), probably to treat a skin disease with the sulfuric waters. However, in the written sources, there is no mention of any possible syphilitic infection affecting the noblewoman, who was famous at that time for her beauty and cultural refinement.

**Figure 11:**
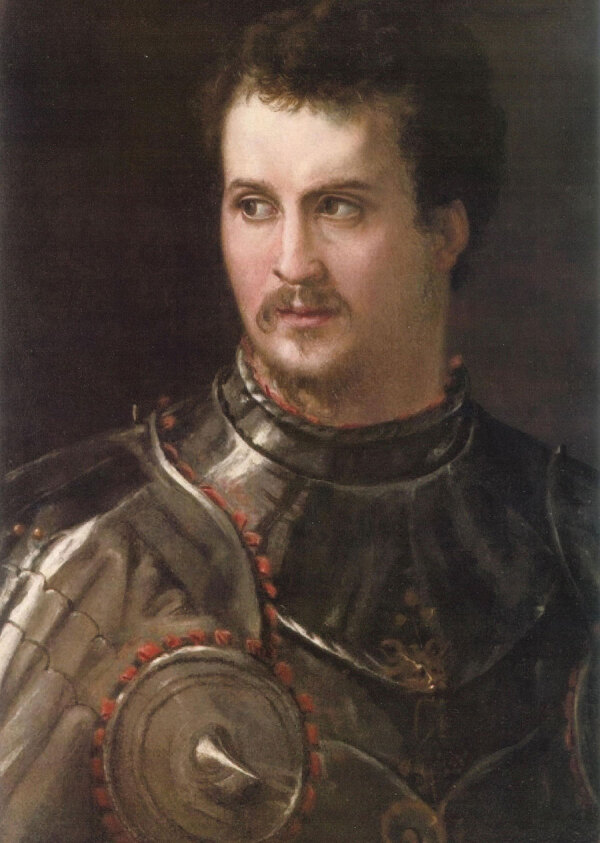
Portrait of Giovanni de’ Medici of the Black Bands, depicted by Francesco de’ Rossi circa 1546–1548. Oil on panel. 25.6 × 18.1 in. (65 × 46 cm). (Florence, Istituti Museali della Soprintendenza Speciale per il Polo Museale Fiorentino, Palazzo Pitti, Galleria Palatina).

It is difficult to speculate on how Maria Salviati contracted the disease, but she is likely to have been infected by her husband Giovanni before his death in 1526, and more probably after the birth of her son Cosimo (June 12, 1519); indeed, the historical sources do not reveal any details about a possible infection of the child, nor do the skeletal remains of the first Grand Duke of Tuscany show any signs of congenital syphilis (*46*). The lifestyle of Giovanni “of the Black Bands” (Figure 11) was characterized by intense sexual extramarital affairs, as witnessed by many documents of the time preserved in the archives of Florence (*24*). On October 20, 1521, Giovanni wrote to his treasurer and lieutenant Francesco Albizi to dispose of war supplies during the military campaign against the French army in Northern Italy: “send me that Greek whore I left in Viterbo” (*47*). On September 22, 1522, during some military actions in the Marche region on behalf of the Pope, Giovanni wrote again to Francesco Albizi, ordering him to kidnap “Lucrezia, courtesan of Rome” and to bring her to him by force (*48*). A considerable series of names of prostitutes frequented by Giovanni are cited in his correspondence: Flora from Padua, Nicolosa “the painted Jewess,” Camilla Orsini, Giulia, Angelica “the Venetian,” Lorenzina “the Greek slave,” Baccia from Rome, Lucrezia nicknamed “Matrema non vole [Mom does not want],” and Paolina (*49*,*50*). The skeleton of Giovanni de’ Medici does not reveal any lesion of syphilis (*26*), probably because he died at the age of 28 years, before the development of the tertiary stage of the disease. 

Extramarital affairs were common among aristocrats (*7*); therefore, syphilis could be considered a disease characterizing the pathocenosis of high social classes. Noblewomen were at risk for contracting sexually transmitted diseases caused by the lifestyle of their husbands, who led an unregulated sexual life characterized by occasional relationships with prostitutes. It was already a well-known fact at the time that syphilis was transmitted sexually (*5*); therefore, some preventive practices were undertaken by the noblewomen, wives, or lovers of the men affected by the disease. Just like Isabella d’Este, wife of Francesco II Gonzaga, Marquise of Mantua, these women sometimes refused to have sexual intercourse with their partners (*7*). Although the disease had no consequences on the reputation of the men, whose sexual conduct was not subject to moral censorship, the situation was different for the women, who tried to hide their sexually transmitted infections (*6*).

## Conclusions

A diagnosis of syphilis for Maria Salviati does not emerge from the historical sources, although the symptoms that manifested in the last years of her life are compatible with a colorectal localization and severe hemorrhages caused by syphilitic infection. In her final years, Maria had a very withdrawn life, possibly to conceal the signs of the illness and certainly for the social complications caused by her recurrent anal hemorrhages (*24*). However, we have no reports that she was marginalized from the ducal court; instead, she was held in high regard as the mother of the reigning duke. In the famous portrait painted by Bronzino during 1542–1543 (Figure 2), she appears veiled and in widow’s clothes, as if to hide the signs of illness. 

The paleopathologic study of Maria Salviati reveals osteolytic and reparative lesions on the skull, which are pathognomonic for syphilis. The diagnosis of syphilis for Maria Salviati enables us to directly observe the gender-based consequences of a pathology to which the Renaissance noblewomen were subjected as a consequence of the sexual conduct of their husbands. The real nature of the disease might have been kept secret at the time for reasons of political opportunity and privacy. A disease that was not a reason of particular shame for sovereigns, princes, and gentlemen, that did not affect their political role and leadership, and was therefore unnecessary to hide, was instead jealously concealed by noblewomen as a “secret illness” that often did not seep outside the private apartments and perhaps did not even reach the attention of the court physicians. This attitude reveals a disparity of perception and of mentality, symptomatic of gender discrimination that was well implanted in the heart of Renaissance society (*6*,*20*). 
